# Circadian Rhythm of Outside-Nest Activity in Wild (WWCPS), Albino and Pigmented Laboratory Rats

**DOI:** 10.1371/journal.pone.0066055

**Published:** 2013-06-07

**Authors:** Rafał Stryjek, Klaudia Modlińska, Krzysztof Turlejski, Wojciech Pisula

**Affiliations:** 1 Institute of Psychology, Polish Academy of Sciences, Warsaw, Poland; 2 Helena Chodkowska University of Management and Law, Warsaw, Poland; 3 Nencki Institute of Experimental Biology, Warsaw, Poland; Simon Fraser University, Canada

## Abstract

The domestication process of the laboratory rat has been going on for several hundred generations in stable environmental conditions, which may have affected their physiological and behavioural functions, including their circadian system. Rats tested in our ethological experiments were laboratory-bred wild Norway rats (WWCPS), two strains of pigmented laboratory rats (Brown Norway and Long Evans), and two strains of albino rats (Sprague-Dawley and Wistar). Rats were placed in purpose-built enclosures and their cycle of activity (time spent actively outside the nest) has been studied for one week in standard light conditions and for the next one in round-the-clock darkness. The analysis of circadian pattern of outside-nest activity revealed differences between wild, pigmented laboratory, and albino laboratory strains. During daytime, albino rats showed lower activity than pigmented rats, greater decrease in activity when the light was turned on and greater increase in activity when the light was switched off, than pigmented rats. Moreover albino rats presented higher activity during the night than wild rats. The magnitude of the change in activity between daytime and nighttime was also more pronounced in albino rats. Additionaly, they slept outside the nest more often during the night than during the day. These results can be interpreted in accordance with the proposition that intense light is an aversive stimulus for albino rats, due to lack of pigment in their iris and choroid, which reduces their ability to adapt to light. Pigmented laboratory rats were more active during lights on, not only in comparison to the albino, but also to the wild rats. Since the difference seems to be independent of light intensity, it is likely to be a result of the domestication process. Cosinor analysis revealed a high rhythmicity of circadian cycles in all groups.

## Introduction

Brown (or Norway) rats (*Rattus norvegicus*) are nocturnal mammals and the bulk of their activity takes place at night. This ecological adaptation, present in the majority of mammalian species, was probably a result of moving into new ecological niches to avoid predators. In the course of evolution, rats developed a number of traits facilitating adaptation to nocturnal life, such as the acute sense of smell which is the dominant sense in rats, specialized vibrissal innervation and representation in the cortex, agouti coat, etc. The ability to dig underground burrows and tunnels provided rats with shelter during the day.

Analysis of the diurnal activity of rats shows that during the day they mostly sleep (approx. 80% of the 12 hours of daylight), while during night they sleep only 30% of time [1]. They also consume around 75% of their daily food intake and a significant proportion of liquids at night [2]. Similarly, mating behaviour [3] and birth of litters are more likely to happen during the dark period [1]. The time of day can also affect rats' cognitive performance. For example, results of learning and memory tests can vary depending on the phase of the diurnal cycle [4].

The main environmental stimulus resetting the phase of the circadian cycle running in the suprachiasmatic nucleus is light [5]–[6]. Light-dark cycle affects a broad set of physiological processes [7]–[9]. Exposing nocturnal rodents to constant illumination results in, among others, lengthening of the period and loss of amplitude of circadian rhythms, reduced activity, disturbances in the circadian cycle of body temperature, decreased food and water intake, as well as impaired learning [9]–[10]. Altogether, 81 genes showing circadian oscillation of expression were identified in 7 different tissues [7].

Exposure to constant dim light or darkness, on the other hand, results in a free-running circadian rhythm that may persist indefinitely with minimal damping. The effect of light is so potent that even brief exposure at night acutely suppresses activity and can induce a phase shift of the circadian cycle [1]. Other important factors are social cues or influence of the group activity that may synchronize slightly divergent internal clocks of individual rats [11]–[13]. As the research on circadian rhythms has been variably performed on singly-housed and group-housed animals, this factor must also be taken into account when discussing mechanisms of circadian cycle of activity [14].

With the circadian cycle being so sensitive to environmental conditions, it can be assumed that the domestication process which has been going on for several hundred generations in uniform breeding conditions, in particular, stable light cycle and intensity, as well as absence of predatory pressure, may have affected a number of physiological and behavioural characteristics of laboratory rats. There is also a possibility that differing conditions in various colonies and multiple forms of directed breeding have produced laboratory rats which are not only very different from their wild counterparts, but also differ in their physiological and behavioural features between themselves [15]–[16]. Because of that, several attempts have been made to establish laboratory lines of rats that are physiologically and behaviourally closer to the wild specimens of the species [17]–[23].

So far, researchers have identified a number of differences between wild and laboratory rats, both in terms of anatomy and behaviour. Apart from different coat colouring and greater body weight, some of the laboratory rats' internal organs tend to be smaller in size [24]–[25]. Wild rats demonstrate higher intra-species aggression [26]–[27] (authors' observations), as well as a greater level of aggression towards humans. The intensity and range of defensive behaviour is also greater in wild rats than in the domesticated rats [18]. Wild rats show stronger aversive reactions to restraint and are more prone to escape [25], [28]. Their anxiety-motivated responses to a novel object are more severe [29]–[34]. Changes in the environment encountered during early development have a more profound effect on their later functioning [35]. They also spontaneously engage in various behaviours (such as climbing), despite lacking previous experience [35]. More recent studies have shown that behavioural differences are also present in such complex behaviours as burrowing (wild rats dig much longer and more intricate tunnel systems) [33] and play-fighting (laboratory rats initiate playful attacks more frequently, and are more likely to use tactics that promoted bodily contact) [36].

In addition, the specifics of response to light in albino animals cannot be ignored [37]–[38]. Albino animals, including rats, lack pigment in the iris and choroid. As a result, the eye has reduced ability for adaptation to light, which may, at very high levels of luminance, lead to photoreceptor degeneration, resulting in impaired vision [39]–[40]. In consequence, light can be a more aversive stimulus for the albino than for pigmented rats [41]. Apart from impaired vision, albino rats have a poorer sense of smell compared to pigmented rats [37]–[38]. They may have slight motor impairments [42], perform worse in spatial tasks [43] and have a different REM sleep pattern [44]. However, our unpublished data show that their highly impulsive and anxiety-driven behaviour may impair their learning ability in complex tasks. They also take much longer to reduce their anxiety and start investigation of objects [32]. Consequently, the behaviour of albino rats (commonly used in research) may differ significantly from the behaviour of both wild rats and the pigmented laboratory rats.

The most popular method of monitoring daily activity of laboratory rats are activity wheels, due to relative ease of use and low cost [1]. However, in wild rats this device may provoke a neophobic responses [31], and consequently – avoidance of the wheel. Furthermore, rats bred for over a century in artificial conditions can differ from their wild conspecifics in their requirement for locomotor activity, as well as in the level of stereotypic activity in the environment of laboratory cage. Studies on motivation have demonstrated that for the albino rats running in the activity wheel creates strong self-reinforcing drive due to release of endorphins [45]. Such repetitive behaviour has no equivalent in the wild and e.g. in zoo animals it is qualified as stereotypy (abnormal, repetitive, unvarying and functionless behaviour) [46]–[48]. Furthermore, locomotion measured this way constitutes only one aspect of rats' overall activity. Time spent on eating, social interaction, etc. remains unaccounted for [49]. In addition, it was found that running in the activity wheel dominates over walking during night, while during the day rats that can move freely prefer walking over running in the wheel [1]. This prompted us to employ an alternative procedure when designing the study, one based on the ethological approach. This type of approach focuses on analysing ecologically valid behaviours of a given species [50]–[53]. In the present study, the measure of rats' activity is the time actively spent outside of shelter (i.e. in the open space of the enclosure).

The circadian cycle of activity, due to its physiological basis and, importantly, availability and relative simplicity of measuring associated parameters, has become a frequent subject of research in behavioural neurophysiology. Opportunities to manipulate the level and frequency of light, feeding times, etc. enable researchers to study a wide range of animal behaviours. At the same time, knowledge of the phases of the circadian cycle and their associated cyclic changes of physiological and behavioural parameters seems indispensable for proper experimental design, as well as for designing optimal breeding conditions.

## Methods

All procedures described in this paper were approved by the 4^th^ Local Ethics Commissions on Animal Experimentation, Warsaw, Poland. All rats prior to experiments were cared for in accordance with the Regulation of the Polish Minister of Agriculture and Rural Development of 10 March 2006 on laboratory animal care.

### Animals

Our sample consisted of 181 rats aged 3 months. As an equivalent of wild Norway rats in this study we used the WWCPS (Warsaw Wild Captive Pisula Stryjek) strain derived in 2006 from breeding pairs obtained from 5 independent colonies of wild rats in Warsaw, Poland [22], [54]–[55]. In order to maintain the population of rats free of domestication features, new rats captured in a variety of locations are systematically added to the colony. The investigated group included 31 WWCPS rats, 40 Brown Norway rats, 32 Long Evans hooded rats, 38 Wistar albino rats and 40 Sprague-Dawley albino rats, roughly matched for sex.

The wild WWCPS rats used in this study belonged to the F2 and F3 generations. The rationale behind using rats from early laboratory bred generations was that a number of researches have reported changes occurring already in early stage of domestication [18], [56]. At the same time, no wild captured rats or their offspring were included in the experiment, as there is no possibility of assessing, let alone controlling conditions in which the animals obtained from the wild developed. Furthermore, a drastic change of environmental conditions may have a profound effect on the rat's level of stress, and consequently on their behaviour during tests, as well as on raising offspring.

The Brown Norway, Long Evans, and Sprague-Dawley rats were sourced from the Mossakowski Medical Research Centre at the Polish Academy of Sciences, while the Wistar rats from the Experimental Medicine Centre at the Medical University of Bialystok, Poland.

Prior to the study, all rats were housed together in our vivarium in standard cages with ad libitum access to food and water. The day/night cycle was 13 h/11 h with the lights-on at 8 AM.

### Equipment

**Figure 1:**
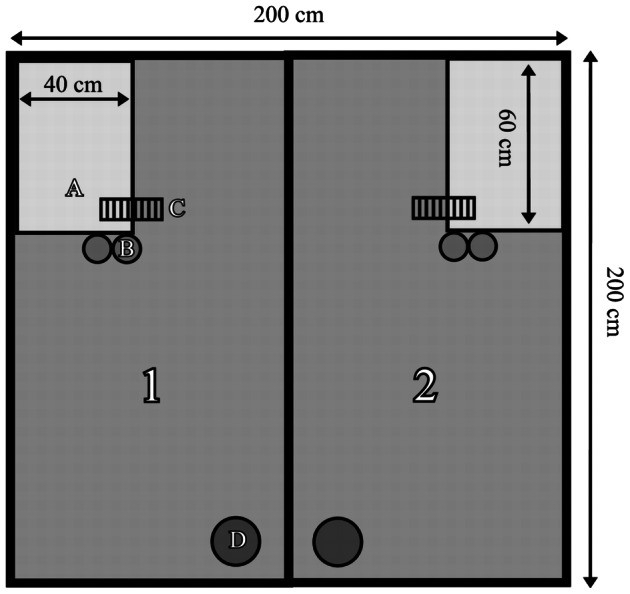
Experimental areas: 1 and 2 – separate boxes for female and male rats; A – two-level shelf; B – water dispensers; C – ladder; D – feeding bowl.

In this study we used two experimental areas with combined dimensions of 200 cm/100 cm/75 cm (Figure 1). The floor of each area was fitted with ceramic tiles. Walls were made of galvanised sheet-plated chipboard. Covers were made of wire mesh mounted on a wooden frame. Each area featured a two-level metal shelf (length 60 cm, width 40 cm, height 40 cm). The bottom level of the shelf was made of metal plate, the upper level was made of transparent plastic plate to enable observation of animals on the lower level. One shelf was installed at 14 cm from the ground, the other at 40 cm. A wire ladder was fixed to each shelf, enabling the rats to climb to either level. The areas also included three water dispensers and a bowl with standard laboratory food. The floor was covered with wood shavings.

The earlier pilot study showed that rats treated the area under the lower shelf as shelter. The choice of that location was probably associated with thigmotaxis, a tendency to move along objects and hide in confined spaces, which is a common phenomenon in rodents, probably driven by predatory pressure [17], [57]. Rats too have a preference for moving and staying in dark and confined spaces or self-dug burrows [58]. Strength of this tendency is assessed in the open field and elevated cross-maze laboratory tests [59].

### Procedure

Rats of one strain were introduced into the living areas. Males and females were placed separately in the two compartments (Figure 1) in groups of 7–10 individuals. Group sizes were as follows (F – female; M – male): WWCPS – 7F/8M, 8F/8M; Brown Norway – 10F/10M, 10F/10M; Wistar – 10F/10M, 8F/10M; Sprague-Dawley – 10F/10M, 10F/10M; Long Evans - 8F/8M, 8F/8M. At the start of the experiment, all animals were 3 months of age. After a 7-day adaptation period (habituation to the experimental conditions), recording of the rats' behaviour commenced. An IR video camera installed above the experimental arena recorded the whole experimental space for 7 successive days - LD (Light-Darkness) experimental conditions. After that time the light in the experimental arena was completely switched off and for the next 6 days, behaviour of the animals was recorded in round-the-clock darkness - DD (Darkness-Darkness) experimental conditions.

The areas were cleaned once a week. The temperature in the experimental room was set at 21°C. Animals had ad libitum access to food and water. The open sections of the experimental arena were lit by fluorescent lamps at 75–100 lx (depending on the location). The day/night cycle in the first phase was set at 13/11 h, with the lights-on at 8 AM. The experimental arena was unlit throughout the second phase of the experiment.

**Figure 2:**
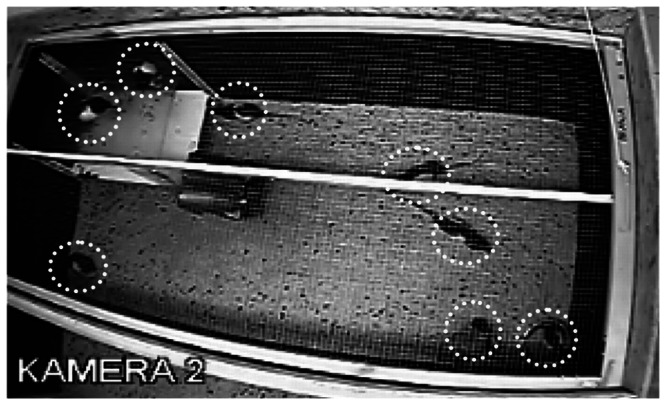
Long Evans rats during their activity outside the nest. An example of the pictures taken by the camera.

The measure of activity was the percentage of rats active outside the nest, i.e. in the visible portion of the arena. All individuals had free access to all parts of the available space and objects. The home environment of the rats included various objects and allowed them to allocate activity in horizontal and vertical plane. The animals sleeping or resting outside their shelter (remaining motionless for at least 5 minutes, lying on their flanks or crouching with their heads lowered and forepaws tucked under the bodies) were also counted. Calculations were made on the basis of every fifth photo from those taken once a minute by a video camera hanging above the experimental arena (Figure 2).

## Results

Although the number of animals in the study was large (N = 181; 5 strains, 4 groups each), they were tested in groups of eight to ten and activity of these groups was assessed. The lack of possibility to assess the behaviour of individual rats was partly offset by the precision of measurements, which were made every 5 minutes for 7 days in each experimental condition and group. For the sake of clarity, outliers (i.e. outlying values in each measured variable) have been excluded using the Grubbs test [60]. For the sample size (N = 4) and the confidence interval of 95%, the outliers were considered to be the results above 1.463 SD in the sample. To account for the fact that comparisons were made between strains, rejections were made on the basis of SD values for each group rather than for the entire sample. The lack of the data for some groups and rejected outliers resulted in differences in size between groups presented in the results of data analysis.

**Figure 3:**
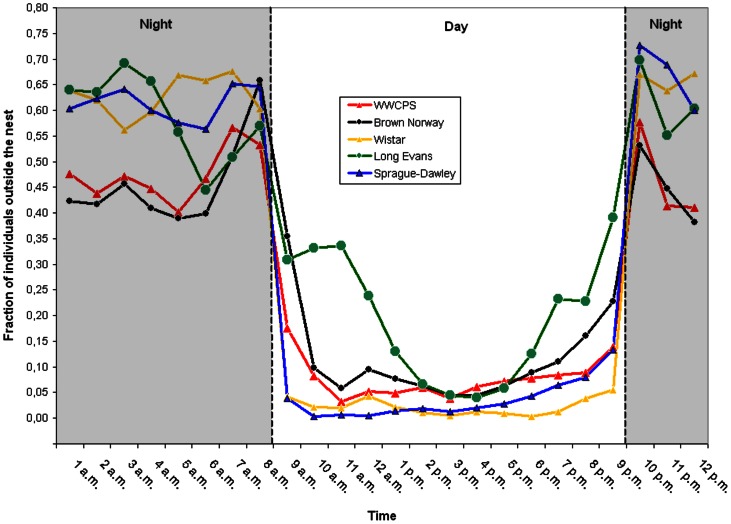
Daily activity of different rat strains.

Results were aggregated by hour, and the mean for each 1-hour period was subjected to further analysis. The final result for a given 1-hour period was the average of measures obtained in 7 successive trial days of the experiment. Figure 3 shows a graphic representation of daily activity for each strain of rats.

**Figure 4:**
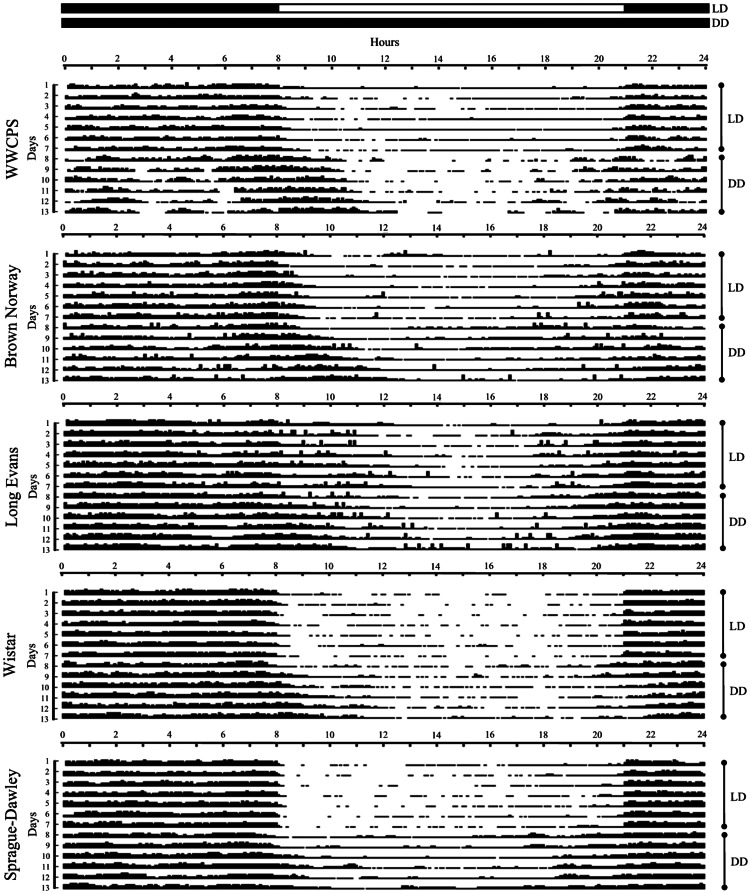
Actogram plots of outside-nest activity rhythm of different strains of rats analysed with 5-min resolution for 13 consecutive days. As indicated by the vertical side bars, a light dark-cycle was in effect for the first 7 days (LD cycle) with lights on at 8 a.m. and off at 9 p.m., whereas for the following 6 days darkness was continuous (DD cycle). The height of bars reflects percentage of rats being active outside the nest.

Circadian activity rhythms were presented as an actogram (Figure 4) [61]–[62]. Each horizontal line represents one day and black vertical bars plotted from left to right represent the outside-nest activity. The height of each vertical bar indicates the accumulated percentage of rats being active outside the nest for a 5-min interval.

### Activity during the day

Mean percentages of rats active during the day were compared in three groups of animals: wild (WWCPS) rats, pigmented laboratory rats (Long Evans and Brown Norway), and albino laboratory rats (Sprague-Dawley and Wistar). Analysis of variance (ANOVA) yielded differences between the groups F(2,17) = 20.20, p<0.000, R^2^ = 70.4%. Post hoc analysis using Games-Howell method for multiple comparisons showed that wild rats were less active during the day than pigmented laboratory rats (Δ = −12.54±2.22, p<0.01). Albino rats demonstrated significantly lower activity than pigmented laboratory rats (Δ = −10.03±2.34, p<0.01). There were no differences between WWCPS and albino rats in this respect.

Comparison between strains using ANOVA confirmed the above assessment F(4,15) = 20.19, p<0.000, R^2^ = 84.3%. But post hoc analysis (Games-Howell method) revealed that it was Brown Norway rats that significantly differ from other strains. Long Evans rats were more active in comparison to Wistar and Sprague-Dawley rats. The results of comparisons are shown in Table 1.

**Table 1:** Comparison between strains in terms of the percentage of rats active outside the nest during daytime.

	WWCPS		BN		LE		Wistar	
	Δ	p	Δ	p	Δ	p	Δ	p
BN	6.01±0.86	**0.047**						
LE	14.06±3.26	0.071	8.05±3.39	0.287				
Wistar	3.21±0.86	0.101	9.21±1.27	**0.017**	17.27±3.16	**0.045**		
SD	−1.8±0.97	0.439	7.81±1.35	**0.020**	−15.86±3.19	**0.050**	1.4±0.58	0.259

Post hoc analysis of ANOVA using Games-Howell method. Δ - mean difference with standard deviation.

Comparison of daily activity of female and male rats yielded no differences (p>0.05).

### Night-time activity

Mean percentages of rats active during the night were compared in three groups of animals: wild rats, pigmented laboratory rats (Long Evans and Brown Norway), and albino laboratory rats (Sprague-Dawley and Wistar). Statistically significant differences were found between the groups – ANOVA F(2,17) = 3.72, p<0.05, R^2^ = 30,5%. But post hoc analysis (Games-Howell method) showed that the differences were only between wild rats and albino rats (Δ = −15.8±3.6, p = 0.012), with WWCPS rats being less active than albino rats. There were no differences between wild rats and pigmented rats or between albino and pigmented rats (p>0.05).

Detailed comparison between strains using ANOVA confirmed the above assessment F(4,15) = 3.44, p<0.05, R^2^ = 47.8%. Post hoc analysis (Games-Howell method) depicted that Sprague-Dawley rats were significantly more active during the night than WWCPS rats (Δ = −17.4±4, p = 0.028). But no other differences were found between the strains in this respect.

Comparison of night-time activity of female and male rats yielded no differences (p>0.05).

**Figure 5:**
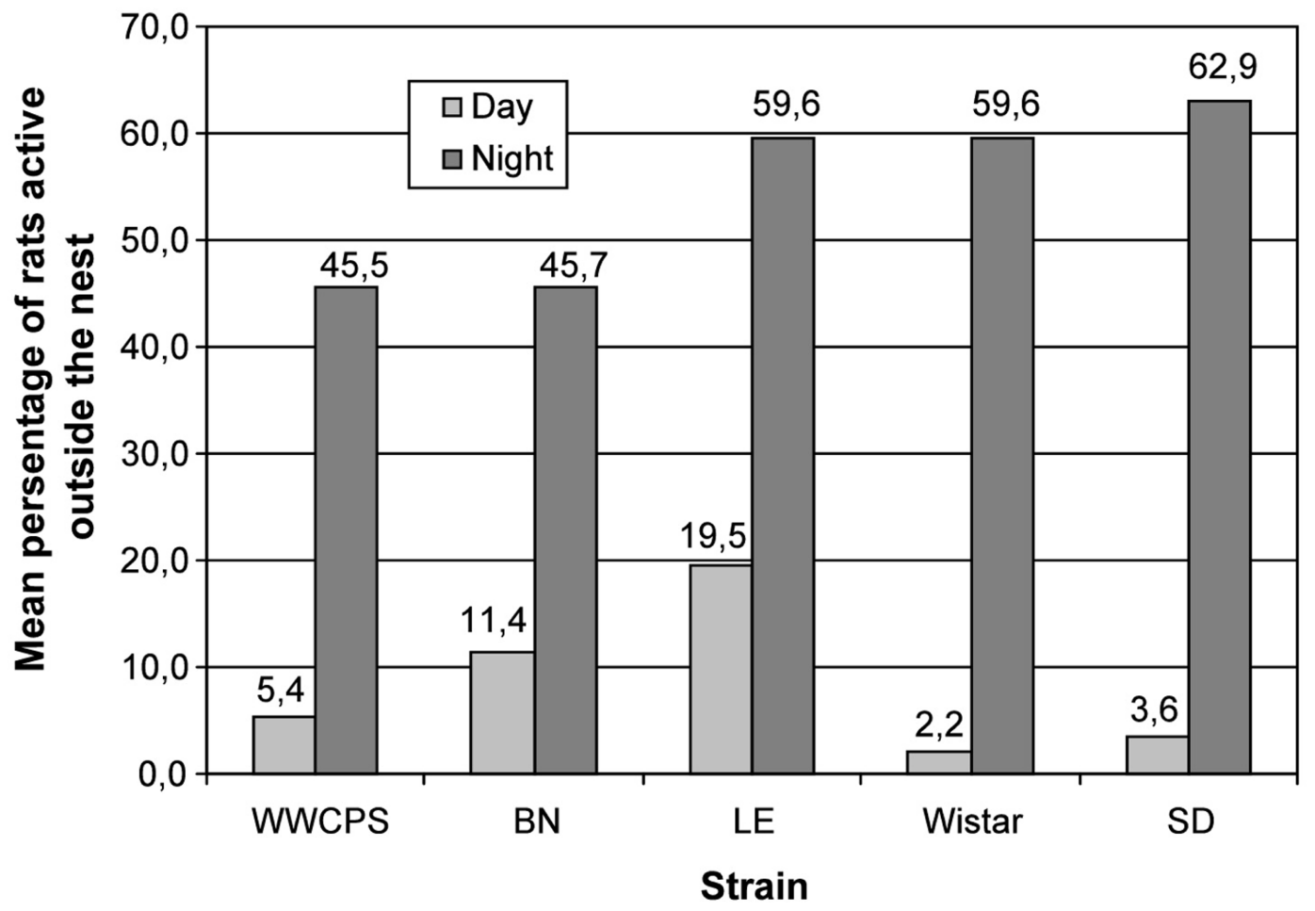
Mean percentage of rats active during the day and night by strain.

A graphical presentation of mean nighttime and daytime activity of individual strains is shown in Figure 5.

### Comparison of day and night mean activity in the LD

Comparisons of activity in LD conditions using Student's t-test revealed differences between day and night activity in each strain. All groups of rats were significantly more active during the night (Tab. 2).

**Table 2:** Comparison between day and night activity (the percentage of rats active outside the nest) in LD.

		LD			
		t	p	Mean	SD
WWCPS	day	−11.142	**0.002**	5.4	1.65
	night			45.5	5.96
Brown Norway	day	−7.011	**0.006**	11.4	1.24
	night			45.7	5.04
Long Evans	day	−3.956	**0.029**	19.5	3.15
	night			59.6	7.06
Wistar	day	−17.794	**0.000**	2.2	0.25
	night			59.6	3.26
Sprague-Dawley	day	−25.468	**0.000**	3.6	0.52
	night			62.9	2.73

Student's t-test.

Mean nighttime activity of rats was subtracted from mean daytime activity in LD conditions to assess an activity change. ANOVA revealed differences between the strains in the variable formed in above way (F(4,15) = 4.13, p<0.05, R^2^ = 52,4%). The change was significantly higher in Sprague-Dawley rats than in WWCPS (Δ = −19.2±4.3, p<0.05) and Brown Norway rats (Δ = −25.0±5.4, p<0.05) - post hoc analysis (Games-Howell method). The analysis yielded no differences between other groups.

### Change in the level of activity at daybreak/nightfall

The magnitude of response to daybreak was measured as the difference in the percentage of rats active outside the nest between the last hour of darkness and the first hour of light. Analysis of variance (ANOVA) revealed differences between the wild, pigmented and albino rats F(2,17) = 6.01, p<0.05, R^2^ = 41.4%. Post hoc analysis (Games-Howell method) conducted for the strains showed that albino rats (Wistar - Δ = −22.1±5.9, p = 0.05 and Sprague-Dawley - Δ = −30.3±5, p = 0.017) demonstrate a greater decline in activity than Brown Norway rats.

A clear increase in activity at the moment when the lights were switched on was observed in all strains. Animals sleeping in the visible section of the arena woke up, started running and initiated social interactions, hid in the nest and ran out of it, etc. None of the groups demonstrated immediate flight to the nest when the lights were switched on.

The magnitude of response to nightfall was measured as the difference in percentage of rats active outside the nest between the last hour of light and the first hour of darkness. Significant differences between strains in the response to nightfall were found using ANOVA F(4,15) = 15.11, p<0.000, R^2^ = 80,1%. Post hoc analysis (Games-Howell method) showed that Brown Norway and Long Evans rats (no significant differences between the two strains) presented lower increase in activity following nightfall than Sprague-Dawley and Wistar rats (no significant differences between the two strains). The results of comparisons are shown in Table 3.

**Table 3:** Comparison between strains in terms of changes in the percentage of rats active outside the nest following nightfall.

	WWCPS		BN		LE		Wistar	
	Δ	p	Δ	p	Δ	p	Δ	p
BN	15.5±5.8	0.168						
LE	15.3±4	0.091	0.2±4.5	1.000				
Wistar	11.5±5.9	0.381	27±6.2	**0.026**	26.8±4.6	**0.029**		
SD	13.5±5	0.165	29±5.4	**0.012**	28.8±3.4	**0.006**	2±5.5	0.995

Post hoc analysis of ANOVA using Games-Howell method. Δ - mean difference with standard deviation.

Qualitative analysis showed that turning lights off was associated with gradual increase in the number of active rats. The animals would start leaving the nest individually or in small groups within a few seconds after darkness fell. However, turning the lights off was not associated with sudden emergence of the majority of rats from shelter (all strains behaved similarly).

### Comparison of the number of rats sleeping outside the nest

**Figure 6:**
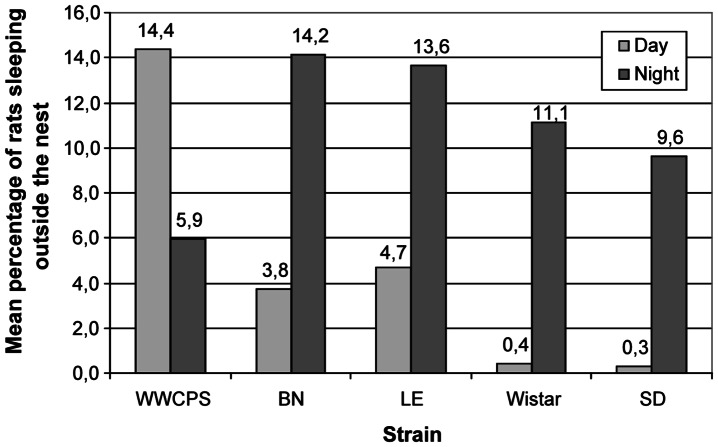
Mean percentage of rats of each strain sleeping or resting outside the nest.

During this study we found that a proportion of animals slept outside the nest and we analysed these results further. The results are presented graphically in Figure 6.

Analysis of variance (ANOVA) showed that there were no differences between the strains in sleeping outside the nest during nights (p>0.05). ANOVA showed differences between strains in sleeping outside the nest during the day F(4,15) = 5.28, p<0.001, R^2^ = 58.5%. However post hoc analysis using Games-Howell method showed no differences between individual groups (p>0.05).

Student's t-test was used to assess the difference in sleeping outside the nest between day and night in LD conditions for each strain – Figure 6. Albino rats slept outside the nest more frequently during the night than during the day (Wistar – t(3) = −19.698, p<0.001; Sprague-Dawley – t(3) = −3.137, p = 0.05). The analysis yieled no differences between other groups in the LD conditions.

### Cosinor analysis of daily changes in activity

Since the experiment was designed to analyse behavioural variability, additional analysis of frequency of cyclic phenomena was conducted. The cosinor analysis using the least squares method was used to fit the cosine curve to time sequences obtained in the study. This method is used to study the cyclic nature of circadian rhythms [63].

In order to determine the free-running period of circadian rhythm the six days of DD cycle (with 5-min resolution) were subjected to time series analysis using chi square periodogram [60]–[61], [64]. The lengths of the internal periods for each strain were presented in Table 4.

**Table 4:** Parameters of the circadian rhythm of activity by groups of animals based on the percentages of animals active outside the nest in three successive time periods.

		Mesor	% rhythmic	Period (hours)	Acrophase (hour)	Acrophase (radians)	Amplitude	Amplitude SE	p value (H0: amplitude = 0)
WWCPS	LD cycle (days 1–7)	23.8	74.2	24.00	02:59	−0.78	25.5	1.16	<.0001
	DD cycle (days 8–13)	27.2	36.5	24.25	n/a	−1.27	18.6	2.05	<.0001
Brown Norway	LD cycle (days 1–7)	27.1	68.1	24.00	03:26	−0.90	23.2	1.22	<.0001
	DD cycle (days 8–13)	27.8	34.1	24.77	n/a	−1.23	16.5	1.33	<.0001
Long Evans	LD cycle (days 1–7)	37.9	82.6	24.00	03:03	−0.80	30.0	1.07	<.0001
	DD cycle (days 8–13)	42.1	77.4	24.00	02:59	−0.78	30.1	1.35	<.0001
Wistar	LD cycle (days 1–7)	28.5	78.7	24.00	02:59	−0.78	39.1	1.53	<.0001
	DD cycle (days 8–13)	35.1	79.5	24.00	03:54	−1.02	33.8	1.45	<.0001
Sprague Dawley	LD cycle (days 1–7)	30.8	78.5	24.00	02:40	−0.70	37.9	1.54	<.0001
	DD cycle (days 8–13)	38.2	76.4	24.13	n/a	−0.59	28.7	1.31	<.0001

Mesor – mean rhythm value; % rhythmic – percentage of variable variance accounted for by rhythm; acrophase – time when the rhythm achieves peak value; amplitude – rhythm amplitude).

The results of the cosinor analysis confirmed rhythmicity of the circadian cycle of activity in all groups of rats in LD and DD conditions (p<0.001) – Table 4. In the round-the-clock darkness WWCPS, Brown Norway, Sprague-Dawley and Wistar rats demonstrated a shift in acrophase (time when the cycle reaches peak value) towards later hours and decreased amplitude of the rhythm (understood as the greatest deviation from the mean value of the rhythm). No shift of acrophase or change in amplitude was found in the Long Evans rats. In addition, WWCPS rats and Brown Norway rats demonstrated a decrease in the percentage of variance accounted for by rhythm in darkness phase. In the remaining groups, the percentage of variance explained by rhythm remained high throughout the study.

In further analyses parameters of waveforms of the activity rhythms in the groups were subjected to comparisons. Analysis of variance (ANOVA) revealed significant differences in mesor values between the strains in both the LD phase (F(4,15) = 9.42, p = 0.001, R^2^ = 71.5%) and DD phase (F(4,11) = 13.4, p<0.000, R^2^ = 83%). Post hoc analysis (Games-Howell method) showed that WWCPS rats differed from Long Evans rats in both light conditions, with wild rats presenting lower values of the variable. Mesor in Long Evans rats was also higher compared to Brown Norway rats in DD conditions and Wistar rats in LD phase. Detailed results of the analysis are presented in Table 5.

**Table 5:** Comparison between strains in mesor of activity rhythm in LD and DD.

		WWCPS		BN		LE		Wistar	
		Δ	p	Δ	p	Δ	p	Δ	p
BN	LD	3.35±2.75	0.747						
	DD	0.6±1.7	0.995						
LE	LD	14.07±1.9	**0.004**	10.73±3.03	0.077				
	DD	15±1.6	**0.003**	14.3±2.1	**0.003**				
Wistar	LD	4.72±1.82	0.203	1.38±2.98	0.988	9.35±2.22	**0.031**		
	DD	8±0.8	0.117	7.3±1.6	0.067	7±1.4	0.057		
SD	LD	7.02±1.79	0.051	3.68±2.96	0.733	7.05±2.19	0.092	2.3±2.13	0.812
	DD	11±2.4	0.058	10.4±2.8	0.062	3.9±2.7	0.627	3.1±2.3	0.701

Post hoc analysis of ANOVA using Games-Howell method. Δ - mean difference with standard deviation.

ANOVA showed no differences between the strains in acrophase of the waveforms of the activity rhythms (p>0.05) in LD conditions. Although ANOVA found differences between the strains in acrophase in DD conditions (F(4,10) = 4.21, p<0.05, R^2^ = 62.7%), post hoc analysis (Games-Howell method) did not confirm that result.

ANOVA revealed differences between the strains in amplitude (F(4,15) = 3.66, p<0.05, R^2^ = 49.4%). The amplitude of waveforms of the activity rhythms in LD was higher in Wistar rats than in WWCPS and Brown Norway rats, as well as higher in Sprague-Dawley than in WWCPS rats. In addition, in DD conditions it was found that the amplitude of the waveforms reached the higher values in Sprague-Dawley rats than in WWCPS and Brown Norway rats, as well as in Wistar higher values than in Brown Norway rats. Detailed results of the analysis are presented in Table 6.

**Table 6:** Comparison between strains in amplitude of activity rhythm in LD and DD.

		WWCPS		BN		LE		Wistar	
		Δ	p	Δ	p	Δ	p	Δ	p
BN	LD	2.3±4.04	0.975						
	DD	2.08±0.92	0.403						
LE	LD	4.5±7.06	0.96	6.8±7.35	0.875				
	DD	11.53±2.82	0.083	13.6±2.78	0.054				
Wistar	LD	13.58±3.48	**0.042**	15.88±4.03	**0.045**	9.08±7.06	0.714		
	DD	15.5±0.75	0.062	17.58±0.54	**0.000**	3.97±2.72	0.64		
SD	LD	12.35±3.1	**0.043**	14.65±3.71	0.055	7.85±6.88	0.782	1.23±3.08	0.993
	DD	10.15±1.44	**0.01**	12.23±1.35	**0.003**	1.38±2.99	0.988	5.35±1.23	0.084

Post hoc analysis of ANOVA using Games-Howell method. Δ - mean difference with standard deviation.

## Discussion

Our analysis of the results identified three groups of rats which differed in activity parameters. As predicted, the behaviour of wild rats differed in some aspects from the behaviour demonstrated by laboratory rats, and there were also differences between the pigmented and albino laboratory rats.

As expected, rats of all groups presented higher outside-nest activity during the night hours in LD conditions. Analyzing the magnitude of difference between day and night activity, we noticed that in LD conditions the difference was higher in Sprague-Dawley than in the wild and Brown Norway rats.

Pigmented laboratory rats showed higher daytime activity than WWCPS rats and they were more active than the albino rats. During night albino (Sprague-Dawley) rats outscored WWCPS rats in that respect. The analysis of difference in time spent sleeping outside the nest between day and night showed that albino rats slept in the open space more frequently during the dark phase of the cycle. During the nighttime-daytime switch, albino rats showed greater decrease in activity than the pigmented (Long Evans) rats. The opposite effect was observed at the nightfall, when the albino rats demonstrated higher increase of activity than pigmented rats.

The pattern of daytime behaviour brings new evidence supporting previous findings on the photophobic responses of the albino rats. Our results suggest that these rats avoid exposure to light by staying in the nest and reducing their daytime activity. This aversion to light is also confirmed by their unwillingness to sleep outside the shelter during daytime hours and sharper inhibition of activity in response to turning the lights on. These results confirm the hypothesis that light is an aversive stimulus for albino rats [37]–[39], [41].

The difference in daytime activity between wild rats and pigmented laboratory rats observed in this experiment seems to be independent of light intensity. Most probably the difference is an unexpected result of the domestication process, reflecting selection for a lower level of anxiety and less pronounced circadian rhythm, as many behavioural experiments were conducted during the time rats usually rest. As experimenters' preferences for daytime activity replaced predators' pressure on animals active during the day, breeding in the laboratory could have gradually reduced influence of circadian oscillations of light-on activity of the laboratory rats. Presumably specimens with relatively high daytime activity were more suitable for conducting experiments during the day and this feature was selected for. The ad libitum and safe access to food removed this mechanism from further evolution and therefore laboratory rats could easily expand their activity to daytime hours. What is more, breeding and keeping conditions in vivaria, forcing rats to stay in cages deprived of dark shelters, may have promoted selective breeding of rats easily tolerating these conditions. In addition, pigmented rats demonstrated no negative response to light such as those presented by albino rats. Such differences are typical for the albino and pigmented specimens of the same species. Moreover, there are reports showing that pigmented laboratory strains of rats may have higher visual acuity than wild rats [65], which can also be explained by adaptation to laboratory conditions.

Lower daytime activity of albino rats could have been also accounted for by the decreased level of locomotor activity, which is a feature of many domesticated animals. However, this assumption is contradicted by the results of activity registration during nighttime. At night, albino rats were more active outside the nest than wild rats. Additionally, when the light was turned off, the increase in activity of the albino rats was larger than that observed in the pigmented strains, which can be explained by the fact that pigmented rats were more active during daytime. It appears that albino rats compensate for their limited activity during the light hours with the increased nighttime activity, which allows them to avoid the aversive light stimulus [41].

The analysis of the circadian rhythmicity of activity outside the nest using the cosinor method showed that all types of rats demonstrated high level of circadian rhythmicity with respect to the investigated behaviour. Following introduction of the round-the-clock darkness, in WWCPS, Brown Norway, Sprague-Dawley and Wistar rats, the peak time of activity was delayed and the rhythm amplitude decreased. In WWCPS, Brown Norway, and Sprague-Dawley rats the changes were accompanied by elongation of the internal circadian period. These findings are consistent with a number of reports claiming that the period of the innate circadian cycle is usually longer than 24 hours and varies from animal to animal, which shows up in the absence of light, that is the regulatory stimulus of the circadian system [1]. However, this feature was not observed in the Long Evans rats.

Interestingly, the cosinor analysis of the circadian rhythm of Brown Norway rats yielded comparable parameters and characteristics to wild rats. A number of variables had similar values, including the decrease in the percentage of variance explained by rhythm.

Detailed analysis of the waveforms of the circadian rhythms formed on the basis of cosinor method revealed differences between the strains in some parameters. In both light/darknes cycle and constant darkness conditions Long Evans rats reached higher mesor values than other pigmented strains. There were no significant differences recorded between the strains in the case of acrophase. This could have been caused by individual differences in the level of activity, that even in the laboratory albino (Wistar) rats are high [66]. Amplitudes of the rhythm were highest in the albino rats, which most probably depended on the unpleasant sensations arising from their impaired accommodation to light. As they had to stay in dark hiding during the day, they were more active during the night.

The study presents the overall outside-nest activity of the rats. However, it would be interesting to conduct an experiment allowing for discrimination between different types of behaviour. It is probable, that the lack of statistical difference in activity levels between some strains is a result of the limitation of the present methodology. Furthermore, it would be worthwhile to test separately housed rats in order to determine the effect of group housing on the circadian rhythm in individual animals. Social synchrony may be an important factor in the process of domestication, in which animals are placed arbitrarily in groups and have not much opportunity to regulate living and social conditions individually.

The findings of the present study may serve as a starting point for further research in this area, looking at more behavioural components and physiological parameters. In the current form they offer clues for the design of experimental procedures, with particular emphasis on light conditions and daily variations in activity patterns.

Besides demonstrating differences between groups, the present study may have methodological significance. Due to strain-dependent differences in responses to light, the best course of action seems to be to conduct behavioural testing in complete darkness or dim lighting conditions, wherever possible. In addition, considering the patterns of diurnal activity, testing times should be carefully selected. While the pigmented laboratory rats are relatively active during the day, the activity of wild rats is at its lowest during daytime. A point to consider by experimenters is whether reversing the day/night lighting cycle in the animal house, so that experimental manipulations occur during the nighttime, would not be the best solution in the case of nocturnal animals. One thing to remember, though, would be that all procedures performed at that time would have to proceed in complete darkness or in dim- or red-lighting conditions, since even brief lighting of the arena may upset the circadian rhythm [64]. Another aspect to bear in mind is the question of laboratory animals' well-being. The results of the present study show that the same lighting conditions, remaining within the spectrum of typical light levels used in laboratories and vivariums may differently influence various strains of the laboratory animals.

In conclusion, differences between laboratory strains of rats observed in our experiment further stress the importance of the careful choice of the strain, as various laboratory strains of rats may differ in some aspects from one another more than they differ from their wild conspecifics. Therefore, choosing a strain without taking into account its specific physiological and behavioural profile, may lead to false conclusions, either due to specific inborn reactions to the experimental procedures or phenotype induced by specific breeding conditions. This means that some inferences from behavioural analysis performed on a given strain of laboratory rat may be fully valid only for that particular strain, and generalisations to the whole population of laboratory rats or the entire species can only be tentative.
